# The Effect of the EMDR Flash Group Technique on Test Anxiety, Traumatic Stress, and Coping Mechanisms in Students Taking a Gap Year

**DOI:** 10.1192/j.eurpsy.2025.1984

**Published:** 2025-08-26

**Authors:** D. Kubilay, A. B. Yaşar, C. Çitil, B. Uğur, S. Kütük, I. G. Sevil, S. Yılmaz, G. Ç. Koban, G. Bedel

**Affiliations:** 1 Psychology, Logos Psychiatry Clinic; 2 Psychiatry, Istanbul Gelisim University, Istanbul; 3 Psychology, Sivas Cumhuriyet University, Sivas; 4 Psychology, Istanbul Bilgi University; 5 Psychology, Sehit Kamil Balkan School; 6 Psychology, Istanbul Ozyegin University; 7 Psychology, Istanbul Gelisim University; 8 Psychology, Y, Istanbul, Türkiye

## Abstract

**Introduction:**

Test anxiety is a widespread issue affecting students’ academic performance and mental health. Students taking a gap year after failing the university entrance exam are particularly vulnerable to traumatic stress and future exam anxiety. An effective group intervention, such as the EMDR Flash Technique, can provide timely support to this group. This technique, an extension of EMDR, involves rapid eye blinking with relaxing imagery during dual stimulation sets, efficiently processing up to five memories per session.

**Objectives:**

The project’s primary objective is to examine how the EMDR Flash Group Technique addresses traumatic stress and exam-related anxiety in gap year students, while promoting adaptive coping strategies. By tackling anticipatory and future-focused stress, the intervention aims to lower the risk of developing mood and anxiety disorders.

**Methods:**

This randomized controlled study will be conducted with a total of 300 students. Participants will be randomly assigned to one of three groups: the EMDR Flash Group Technique, a single-session psychoeducation seminar, and a waitlist control group. Symptoms will be measured at pre-treatment, post-treatment, and follow-up. The assessment tools include the Socio-demographics Scale, Test Anxiety Inventory, Coping with Stress Scale, and the Impact of Event Scale. This study will address the following hypotheses: H1: EMDR Flash Group Technique will decrease the level of test anxiety. H2: It will decrease traumatic stress based on previous test experiences. H3: It will develop functional coping mechanisms.

**Results:**

A significant reduction in exam anxiety levels is expected in the EMDR Flash Group participants, supporting its effectiveness in managing stressors and preventing the development of anxiety disorders. Additionally, there will be a decrease in traumatic stress symptoms (re-experiencing, avoidance, and hyperarousal), demonstrating its effectiveness as an early intervention for trauma-related disorders. Participants are expected to develop more functional stress management skills, emphasizing the technique’s role in reducing dysfunctional coping mechanisms and enhancing resilience. Overall, the EMDR Flash Group Technique will demonstrate its effectiveness as an early intervention model for managing anxiety and preventing the progression of related psychological issues.

**Image 1:**

**Figure:**
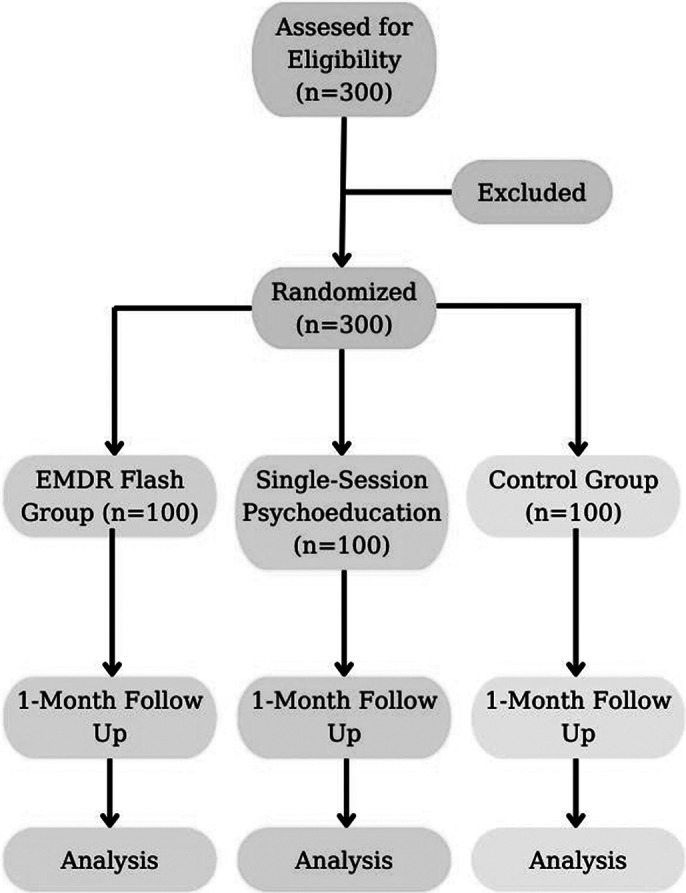

**Image 2:**

**Figure:**
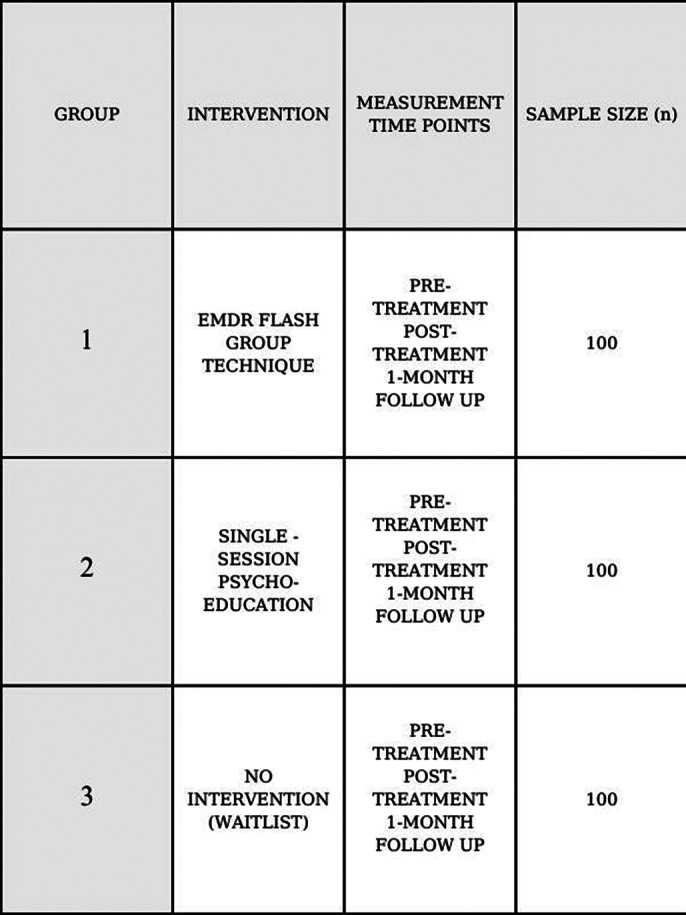

**Conclusions:**

Unlike traditional EMDR protocols that focus primarily on past traumas, our approach uniquely integrates past, present, and future-focused elements, creating a holistic framework for managing both past traumatic experiences and anticipatory anxiety about future challenges. By employing a group-based format, this protocol not only offers the potential for a more scalable and efficient intervention, but also provides a deeper understanding of how EMDR can be adapted to address psychiatric disorders in youth.

**Disclosure of Interest:**

None Declared

